# Late Development of a Bronchocutaneous Fistula due to an Epicardial Cardioverter-Defibrillator Lead

**Published:** 2015-04-03

**Authors:** Vasileios Patris, Orestis Argiriou, Niki Lama, Haris Georgiou, Constantine Halkias, Christos Charitos

**Affiliations:** 1 *Heart and Chest Hospital, Liverpool, UK.*; 2 *Medical School, University of Ioannina, Ioannina, Greece.*; 3 *Evangelismos General Hospital, Athens, Greece.*; 4 *North Middlesex University Hospital, London, UK.*

**Keywords:** Defibrillator, implantable, Pacemaker, artificial, Fistula, Hemoptysis

## Abstract

Cutaneous complications caused by a pacemaker or defibrillator are widely documented, but the development of a bronchocutaneous fistula has never been described before. We report the case of a 79-year-old man who was admitted to our hospital because of a seemingly superficial cutaneous infection, externalized defibrillator leads, and hemoptysis. Bronchoscopical investigation proved the existence of the fistula, which connected the epicardium, the left main bronchus, and the aforementioned site of skin infection. The patient refused an operation for the complete removal of the epicardial defibrillator and was treated conservatively. This case demonstrated that the long-term presence of foreign bodies in the epicardium may cause serious complications.

## Introduction

Currently, there are over 3 million patients within the United States with implanted cardioverter-defibrillator devices, and approximately 400000 devices are implanted each year.^1^ These devices are inserted to prevent significant arrhythmias, which can lead to sudden death.^2^ Implantable cardioverter-defibrillators have been associated with low morbidity.^3^ Complications do arise, and they mainly include bleeding, lead fracture, false discharges, failure of termination, early generator failure, and pericardial constriction.^3^ Another adverse complication is that the insertion of a foreign material within the body, such as a pacemaker device or epicardial leads, can carry a certain risk of infection. The following case describes a very rare complication of a first-generation defibrillator implanted during cardiac surgery (coronary artery bypass grafting), which is the formation of a bronchocutaneous fistula. 

## Case Report

A 79-year-old man presented to our hospital with a 15-day history of hemoptysis, general malaise, night fever, and weight loss. The patient had no known respiratory comorbidities, but had an extensive cardiac history of a myocardial infarction in 1992, leading to poor left ventricular (LV) function (ejection fraction of 35%). Due to the severity of the infarct, an urgent coronary artery bypass grafting to his left anterior descending, posterior descending artery, and first obtuse marginal was scheduled, that same year. During the procedure, a pacemaker-defibrillator was fitted with leads directly positioned on the epicardium via a series of flattened coils. The surgeon’s decision to insert a pacemaker-defibrillator device was secondary to the poor LV function. It was noted that the patient had no early postoperative complications. Two years after the procedure, the pacemaker device was extracted under local anesthetic as per protocol, but four epicardial leads were difficult to extract and were left in the thorax, as the patient wished no further surgical intervention.

**Figure 1:**
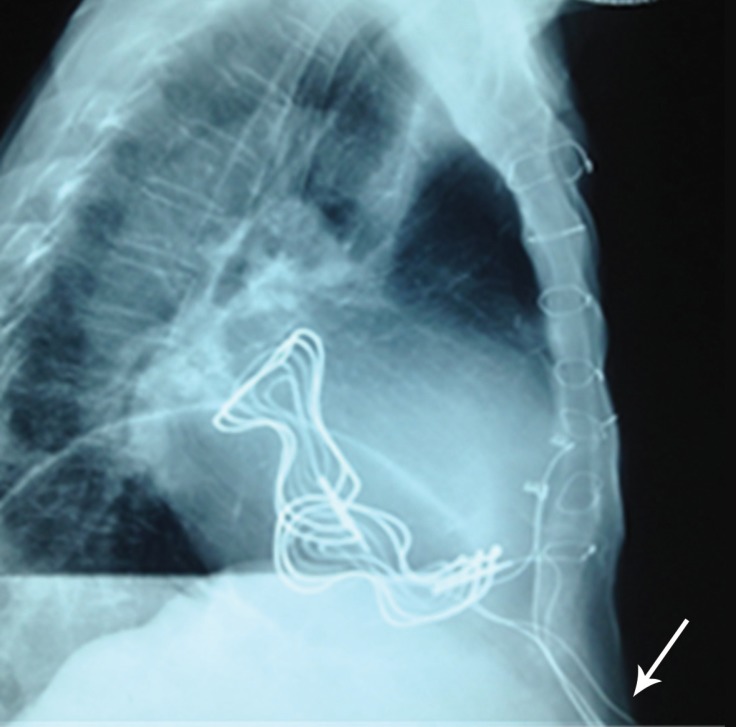
Lateral chest radiograph, showing the externalization of the leads (arrow)

**Figure 2:**
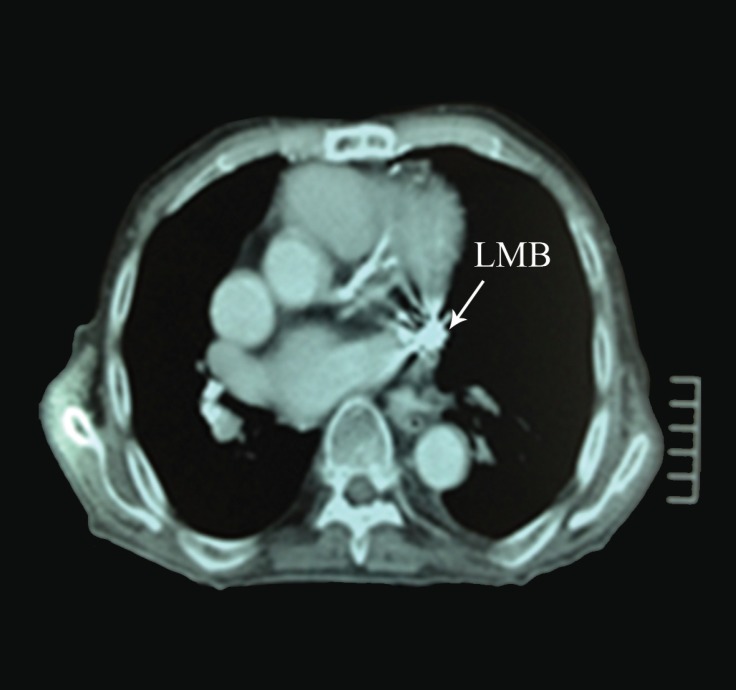
Computed tomography, showing the epicardial patch electrode in contact with the LMB (left main bronchus)

The patient was admitted to our hospital 18 years later, with a 15-day history of hemoptysis, general malaise, fever, and weight loss. Upon examination, a 3 cm × 5 cm circular aperture was noted approximately 2 cm below the xiphisternum; ulcerated tissue was protruding from the aperture and four externalised pacing leads were visible, accompanied by mild erythema. Physical examination of the heart, lungs, and abdomen provided no further findings. During the admission, it was also revealed by the patient that the fistula-like structure had presented initially 2 years after surgery, with continuous secretory fluid drainage from then. Laboratory findings demonstrated neutrophilia of 80.9 and raised C-reactive protein. Bronchoscopy showed minor hemorrhage at the carina and at the entrance of the left upper bronchus. X-ray and computed tomography (CT) scans of the chest indicated that the leads were in contact with the left main bronchus and also confirmed the externalization (Figures 1 and 2). 

 Secondary to those clinical and bronchoscopical findings, the patient underwent a fistulography, which proved that the fistula was connecting the left main bronchus to the epicardium. Additionally, further proof was provided as, after contrast administration via the cutaneous site, the patient had a severe episode of respiratory distress with coughing and shortness of breath. Cultures of the pus identified the presence of Morganella morganii, Enterococcus faecalis, and Klebsiella pneumoniae. Therapeutic plan was based on the administration of intravenous antibiotics such as Ampicillin-Sulbactam and Moxifloxacin, due to the suspected infective process. However, no symptom resolution was achieved and the debridement of the exit site of the leads was performed. Anti-platelet therapy was suspended, which led to the cessation of the hemoptysis. According to the patient’s wishes, no further surgical interventions were attempted (removal of the epicardial device and closure of the fistula) due to the high operative risk. The patient was treated conservatively, and was discharged shortly afterward. Although frequent follow-up was advised, the patient showed up two years later, at which point a chronic, local inflammation of the exit site of the leads was observed. Local care and sterilization were applied at the hospital. The patient also reported frequent upper respiratory system infections (with most probable entrance site being the exit site of the leads), which were treated conservatively with antibiotic administration by his general physician. 

## Discussion

Since 1985, 40000 patients have had surgical implantations of an epicardial cardioverter-defibrillator as the best therapy to prevent death due to arrhythmogenic ventricular cardiomyopathy.^4^ Currently, the use of epicardial cardioverter-defibrillator is chosen only in cases of the insertion of prosthetic tricuspid valves (in which transvenous endocardial cardioverter-defibrillator lead implantation is contraindicated), in pediatric cases (in which the patient’s size does not allow the insertion of transvenous leads),^5^ or in cases where adequate defibrillation thresholds cannot be reached with the transvenous approach. Also, patients on hemodialysis, patients with an occluded upper venous system, or patients with previously implanted transvenous devices should be treated with the use of an epicardial cardioverter-defibrillator.

A significant number of complications following implantable cardioverter defibrillator (ICD) implantation have been described, such as device pericardial constriction, pericardial tamponade, pocket hematoma, seroma, wound infection and dehiscence, device migration, lead fracture, right ventricular perforation, pneumothorax, hemoptysis, and bronchoenteric fistulas.^6^

This report details another possible complication of an automatic ICD patch left in the pericardium of a patient who had received an implantable defibrillator 18 years previously: a bronchocutaneous fistula. The current practice has moved away from the use of epicardial patches in favor of direct epicardial contact leads. 

A sternobronchial fistula after coronary surgery due to retained epicardial temporary pacing wires has previously been described;^7^ nevertheless, our literature review revealed no reports of a bronchocutaneous fistula as a complication due to an epicardial defibrillator 18 years after implantation. We believe that chronic low-grade infection of the defibrillator system may have contributed to this complication. 

Despite the fact that this intervention leads to relatively high morbidity and mortality rates, complete patch removal should be chosen as a therapeutic plan. As with any intervention, a number of factors should be taken into consideration before employing a management plan. In the case of our patient, his adhesions, age, and concomitant diseases were factors that rendered the removal extremely difficult with an associated high mortality rate.

## Conclusions

The long-term presence of foreign bodies in the epicardium may lead to serious, even fatal, complications. Re-do surgery, in order to achieve the complete removal of any hardware causing these complications, proves to be the gold-standard treatment. However, conservative treatment should be considered in cases with high perioperative risk.

## Notes:

***This paper should be cited as: ***Patris V, Argiriou O, Lama N, Georgiou H, Halkias C, Charitos C. Late Development of a Bronchocutaneous Fistula due to an Epicardial Cardioverter-Defibrillator Lead. J Teh Univ Heart Ctr 2015;10(2):106-108.
